# Bronchial asthma and COPD due to irritants in the workplace - an evidence-based approach

**DOI:** 10.1186/1745-6673-7-19

**Published:** 2012-09-26

**Authors:** Xaver Baur, Prudence Bakehe, Henning Vellguth

**Affiliations:** 1 Institute for Occupational and Maritime Medicine, University Medical Center Hamburg-Eppendorf, Seewartenstr. 10, 20459, Hamburg, Germany

**Keywords:** Work-related asthma, Occupational asthma, Occupational COPD, RADS, Irritant-induced asthma

## Abstract

**Background:**

Respiratory irritants represent a major cause of occupational obstructive airway diseases. We provide an overview of the evidence related to irritative agents causing occupational asthma or occupational COPD.

**Methods:**

We searched MEDLINE via PubMed. Reference lists of relevant reviews were also screened. The SIGN grading system was used to rate the quality of each study. The modified RCGP three-star system was used to grade the body of evidence for each irritant agent regarding its causative role in either occupational asthma or occupational COPD.

**Results:**

A total of 474 relevant papers were identified, covering 188 individual agents, professions or work-sites. The focus of most of the studies and the predominant diagnosis was occupational asthma, whereas occupational COPD arose only incidentally.

The highest level assigned using the SIGN grading was 2+ (well-conducted systematic review, cohort or case–control study with a low risk of confounding or bias). According to the modified RCGP three-star grading, the strongest evidence of association with an individual agent, profession or work-site (“**”) was found for 17 agents or work-sites, including benzene-1,2,4-tricarboxylicacid-1,2-anhydride, chlorine, platinum salt, isocyanates, cement dust, grain dust, animal farming, environmental tobacco smoke, welding fumes or construction work. Phthalic anhydride, glutaraldehyde, sulphur dioxide, cotton dust, cleaning agents, potrooms, farming (various), foundries were found to be moderately associated with occupational asthma or occupational COPD (“*[+]”).

**Conclusion:**

This study let us assume that irritant-induced occupational asthma and especially occupational COPD are considerably underreported. Defining the evidence of the many additional occupational irritants for causing airway disorders will be the subject of continued studies with implications for diagnostics and preventive measures.

## Introduction

Bronchial asthma and chronic obstructive pulmonary disease (COPD) are common conditions and are the dominating obstructive airway diseases in the general population.

### Work-related asthma (WRA) including irritant-induced occupational asthma (OA)

Occupational asthma is defined as a chronic inflammatory disorder of the airways with recurrent episodes of coughing, wheezing, chest tightness, dyspnea, shortness of breath at rest, and reversible airflow limitations caused by a particular occupational environment [1-3].

**Figure 1:**
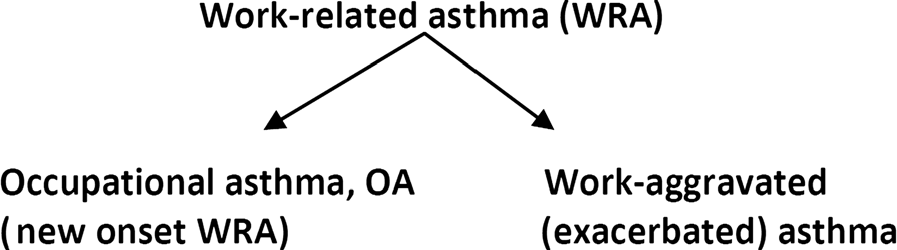
**Work-related asthma is dividedinto occupational asthma andwork-aggravated asthma.**

The available epidemiological and comparative studies and reviews provide evidence that occupational agents cause 5 – 25% of all asthma cases [1,4-23]. Besides these evident occupational asthma (OA) cases, there is probably an even larger population of sufferers of work-aggravated asthma [24-26]. The latter population shows an objective worsening of pre-existing asthma or non-occupational asthma that develops in parallel with causative conditions encountered in the workplace (Figure 1).

**Figure 2:**
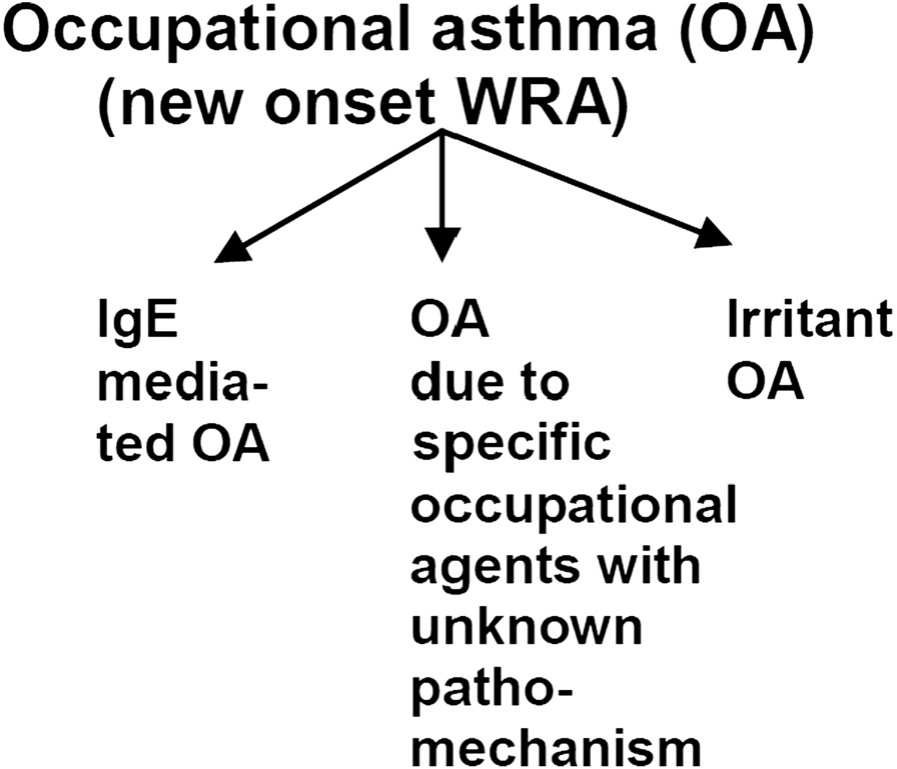
Subgroups of OA.

Occupational agents eliciting bronchial asthma, i.e. OA, comprise occupational allergens, with their well-defined etiological role and IgE-mediated pathomechanism, as well as occupational agents with unknown pathomechanisms and occupational respiratory irritants, mainly representing low molecular weight chemicals (LMW; <5000 Daltons) causing irritant-induced OA (Figure 2). The latter agents may also elicit occupational COPD (see chapter 1.2) and include chlorine, acids, welding fumes, as well as isocyanates. The etiological role of such low molecular chemicals has not yet been completely clarified, primarily because of the lack of specific diagnostic tests.

There is sparse data available on causes and frequencies of irritant-induced COPD and work-aggravated asthma. Therefore, this work focuses on irritant-induced OA.

There is increasing evidence that irritant-induced OA can be further subdivided into three subcategories as outlined in Table 1[27-29].

**Table 1:** Subcategories of irritant-induced OA

**Subcategories of irritant-induced OA**	**Exposure concentration**	**Duration of exposure**
Reactive airways dysfunction syndrome (RADS)	Extremely high, > OEL	≤1 day
Not so sudden onset of irritant-induced OA	Moderate, around OEL	>1 day <4 months
Low dose irritant-induced OA	Low, below OEL	>4 months

OA = Occupational asthma, OEL = Occupational Exposure Limit.

Many case reports, case series and a few cross-sectional studies demonstrate that a single short-term accidental massive exposure or several short-term high-level exposures to a respiratory irritant can cause asthma within 24 hours without a latency period. Brooks et al. [30] defined this disorder as “reactive airways dysfunction syndrome” (RADS). This term was later extended to irritant-induced OA from multiple, somewhat lower, exposure incidents with a less sudden onset that were also shown to cause this disorder [27,31-36].

Furthermore, there is evidence that a susceptible subgroup of subjects mainly atopics with non-specific bronchial hyperresponsiveness (NSBHR) suffering from irritant-induced OA, is also affected by chronic exposures to relatively low concentrations of irritant gases, fumes or aerosols [27,37,38]. This disorder has been called “low-dose irritant asthma” (or “low-dose RADS”). Corresponding studies indicate respiratory effects including asthma from mainly chronic or repeated exposure to a single irritant or a mixture. Demonstrably causative concentrations of a particular irritant are often below their occupational exposure limits (OELs) or permissible exposure limits (PELs). Such irritant examples include swine confinement facilities [39,40], exposures to cleaning agents [12,41], solvents, ozone, endotoxin, formaldehyde, quaternary ammonium compounds, chlorine, bisulfite and SO_2_, or acid mist [36,37,42-44], diesel exhaust [10,45,46], fumigant residues [47], dusts in the textile paper, mineral fiber or construction industries or in mines [48-51], as well as a proportion of cases of potroom asthma [52] and meatwrappers' asthma [53]. Asthma in cold-air athletes may also be relevant [54,55]. A previous summary of the literature on respiratory effects from asthma due to irritants below their OELs/PELs is available [56]. Many of the earlier exposure limits have been lowered repeatedly in the light of subsequent clinical or epidemiological findings on their respiratory effects. Other limits remain obstinately high given their known irritative effects and/or that they are based on sparse data [56]. Accordingly, adherence to OELs/PELs does not preclude the onset of WRA in susceptible subjects.

The broader definition of these disorders (as used in the legal definition in Germany) includes all irritant-induced obstructive airway diseases irrespective of their causative concentrations and reversibility, i.e. irritant-induced occupational asthma as well as COPD.

### Frequency of OA

OA has become the most prevalent occupational lung disease in developed countries [57,58] and it is one of the most frequent diagnosis among occupational diseases in general [59]. The annual incidence of OA is in the range of 50 per million with extremes up to 250 per million workers and more than 1,300 per million in specific workplaces [57,60]. As already mentioned there is evidence that occupational agents cause 5 - 25% of of all asthma cases. However, complete registries of OA do not exist and therefore the true frequency of the disease is unknown. Ameille et al. [61] and Fernández-Nieto et al. [59] stated that OA is underestimated among occupational diseases, because many OA cases are not subjected to appropriate diagnostic tests.

Irritant-induced OA is reported to occur in approximately 5 -18% of all OA cases, being the second most common form of OA after allergic OA [36,62].

### Chronic obstructive pulmonary disease (COPD) due to occupational exposure

The diagnosis of COPD is based on chronic productive cough, airflow limitation that is usually not fully reversible, and a progressive, abnormal inflammative response of the lungs mostly caused by long-term smoking and by other noxious particles or gases [1].

During ongoing causative exposures (e.g. smoking particles, droplets and/or gases), airflow limitation is usually progressive and associated with an abnormal inflammatory response of the lungs. Patients with COPD have greater number of neutrophils and alveolar macrophages in bronchoalveolar lavage fluid than healthy non-smokers [63]. Sites of emphysema, which are frequently found in COPD patients, contain large numbers of lymphocytes, and the extent of lymphocyte accumulation correlates with reduction of FEV_1_.

In their summaries of the literature, Hnizdo et al. [64], Trupin et al. [65] and Balmes et al. [1] found an occupational contribution in about 15% of COPD cases.

Occupational COPD is identified on epidemiological basis, by observing increased frequencies of COPD among certain working groups [66], e.g. in construction workers [2]. Some occupational exposures (e.g. welding fumes, aluminium, potroom fumes, and cadmium) may cause COPD associated with emphysema [67,68].

At later stages of OA, the condition of some subjects does not improve over weekends or during holidays and coincides with symptoms of COPD patients. This observation also applies to non-occupational obstructive airways diseases [69,70] and indicates that a group with changing diagnoses as well as with some overlap between OA and occupational COPD, does exist [66,71-73].

## Background and objective

WRA and occupational COPD are serious and sometimes fatal diseases, which can lead to ill health, inability to work and lost productivity [1,25,74-76]. They represent a huge economic burden to the society. For details see Additional file 1: Online Supplement “Economic burden”.

The objective of this study is to summarize present knowledge on respiratory irritants causing obstructive airway diseases in humans in the occupational setting and to provide a rating of the strength of evidence for each irritant which has not been previously available.

## Methodology

A systematic review of the literature on occupational irritant-induced OA and occupational COPD due to occupational irritants was conducted. We considered asthma-inducing irritating agents as well as those reported to cause occupational COPD and related disorders, where obstructive ventilation patterns were demonstrated in clinical investigations, cross-sectional studies, cross-shift and/or in long-term exposure studies. Irritating gases mainly occurring in the general environment, such as ozone, and inorganic dusts, including silica, talcum, silicates and other fibers known to cause pneumoconioses, were not considered even though exposure to them is frequently associated with mixed ventilation patterns.

### Definitions used

Occupational COPD: chronic bronchitis symptoms and non-reversible airflow limitation due to particular occupational environment (if lung function data was available; otherwise, clinical diagnosis as given by the authors is cited).

Occupational asthma: episodes of shorteness of breath due to particular occupational environment and reversible airflow limitation (if lung function data was available; otherwise, clinical diagnosis as given by the authors is cited).

Obstructive ventilation pattern: we applied reference values of FEV_1_/FVC from Brändli, Schindler et al. 2000 [77].

### Information sources and selection criteria

#### Occupational respiratory irritants

To identify the evidence of irritants of the respiratory tract, all agents denoted as “may cause respiratory irritation” by the phrase H335 (previous code R37) and “may cause allergy or asthma symptoms or breathing difficulties if inhaled” H334 (previous code R42) [78] and/or as “irritants” by American Conference of Governmental Industrial Hygienists [79] were initially listed [80]; later this list was compared with results of our database search (see below).

Database search.

We searched for publications reporting investigations exclusively in humans (i.e. animal or in-vitro research was excluded). To be included, the publications had to deal with subjects occupationally exposed to airway irritants.

MEDLINE®-Database was searched with PubMed® from its inception up to December 2007 with the following medical subject headings (MeSH) combinations for each single agent:

“*Agent*”[MeSH] AND “Humans”[MeSH] AND ((“Asthma”[MeSH] OR “Asthma/chemically induced”[MeSH] OR “Asthma/immunology”[MeSH]) OR “Pulmonary Disease, Chronic Obstructive”[MeSH] OR “Lung Diseases, Obstructive/*chemically induced”[MeSH] OR “Respiratory Function Tests”[MeSH]) AND (“Accidents, Occupational”[MeSH] OR “Occupational Exposure”[MeSH] OR “Occupational Diseases”[MeSH] OR “Occupational Diseases/chemically induced”[MeSH])).

If more than 20 publications per agent were found, the search was more specified:

“*Agent*”[MeSH] AND “Humans”[MeSH] AND (“Cohort Studies”[MeSH] OR “Case–control Studies”[MeSH] OR “Case–control Studies”[All Fields] OR “Longitudinal Studies”[MeSH] OR “Longitudinal Studies”[All Fields] OR “Cross-Sectional Studies”[MeSH] OR “Cross-Sectional Studies”[All Fields] OR “Epidemiologic Studies”[MeSH] OR “Epidemiologic Studies”[All Fields] OR “Case Reports”[Publication Type] OR “Meta-Analysis”[MeSH] OR “Meta-Analysis”[All Fields]) AND “adverse effects”[Subheading] AND ((“Asthma”[MeSH] OR “Asthma/chemically induced”[MeSH] OR “Asthma/immunology”[MeSH]) OR “Pulmonary Disease, Chronic Obstructive”[MeSH] OR “Lung Diseases, Obstructive/*chemically induced”[MeSH] OR “Respiratory Function Tests”[MeSH]) AND (“Accidents, Occupational”[MeSH] OR “Occupational Exposure”[MeSH] OR “Occupational Diseases”[MeSH] OR “Occupational Diseases/chemically induced”[MeSH])).

#### Reference list screening

We also considered references in the identified already existing 13 systematic reviews or overviews of causes of work-related asthma or COPD and tried to combine results of both approaches.

### Occupational diseases statistics

Further, we considered the following occupational diseases statistics based either on statutory surveillance or registration systems: SWORD 1994–1997 [81-83]; SHIELD 1993 [74]; SORDSA 2001 [84]; SENSOR 2003 [85]; Dokumentation der Berufskrankheiten 2007 (BK-DOK) [86].

### Conditions

Four different conditions were accepted for inclusion:

1. Irritant-induced OA including RADS. Asthma caused by single or multiple occupational exposures to airway irritants; de novo irritant-induced OA. Asthma within 24 hours without a latency period caused by short-term high-level exposures to a respiratory irritant known as acute irritant-induced asthma, or as RADS [30].

2. Work-aggravated (exacerbated) irritant-induced OA. Pre-existing or concurrent asthma worsened by work factors [24]. Subjects with work-related asthmatic symptoms, if not differentiated whether new-onset or work-aggravated.

3. Occupational COPD. On epidemiological basis, identified by observing increased frequencies of COPD among certain working groups [66].

4. Obstructive ventilation pattern. Studies about irritant agents, where obstructive ventilation patterns in occupational settings were reported.

### Methodological selection criteria

Publications with one of the following study designs were included: Systematic reviews of cohorts, case–control or cross-sectional studies, cohort studies (prospective/retrospective), case–control studies, cross-sectional studies, surveys.

Non-analytic1^a^ studies (i.e. case series, follow-up of cases or case reports) were only included when for an agent no studies with one of the above mentioned designs had been identified.

Publications were included when they met any of the following criteria: examining the frequency of irritant-induced OA or asthmatic work-related symptoms in occupationally exposed groups or individuals, reporting the causative role of the specific agent or mixture of agents for irritant-inducing WRA or COPD.

Studies were included when they applied any of the following diagnostic tools: description of work-related asthmatic symptoms (questionnaire), lung function test (LFT), testing for non-specific bronchial hyperresponsiveness (NSBHR) by means of methacholine, histamine or other pharmacological agents, serial spirometry or expiratory peak flow (PEF) monitoring or supervised exposure testing in the workplace, challenge with the help of lung function measurements (SFT), specific inhalation challenge testing (SIC), clinical diagnosis of OA by an expert (occupational or pulmonary physician), and exposure to an irritant agent.

Publication period: No restriction for publication dates were made, last updates were between 5^th^ and 15^th^ June 2012.

Language: English, German, Spanish, Italian or French.

Methodological studies, e.g. on effects of study design and subsequent procedures, and studies with non-occupational disorders were excluded. Publications about occupational agents which do not have an irritant effect on the respiratory tract (e.g. about IgE-sensitizing agents) or with unrelated issues (e.g. studies on immunological questions) were also excluded.

### Assessment of study quality

The principal study characteristics and study results were systematically extracted using an extraction sheet (see Additional file 2: Table S1A of online supplement “Methodology”).

We assessed study quality with the help of a check list (see Additional file 2: Table S1B of online supplement “Methodology”). The evidence level of each study was graded according to the revised Scottish Intercollegiate Guidelines Network (SIGN) grading system [87]. Since population-based randomized assignment to different levels of irritant exposure are unethical, no randomised controlled trials (RCTs) could be expected on this topic and, thus, no level 1 evidence (as defined by the revised SIGN grading system) [87] would be available. In order to achieve more differentiation among lower evidence grades, we modified the SIGN grading system and added an additional grade (3+) (see Additional file 2: Table S1C of online supplement “Methodology”).

Details of the modified RCGP [88] grading system are given in the online supplement “Methodology”, Additional file 2: Table S1D.

## Results

### Overview on publications retrieved

**Figure 3:**
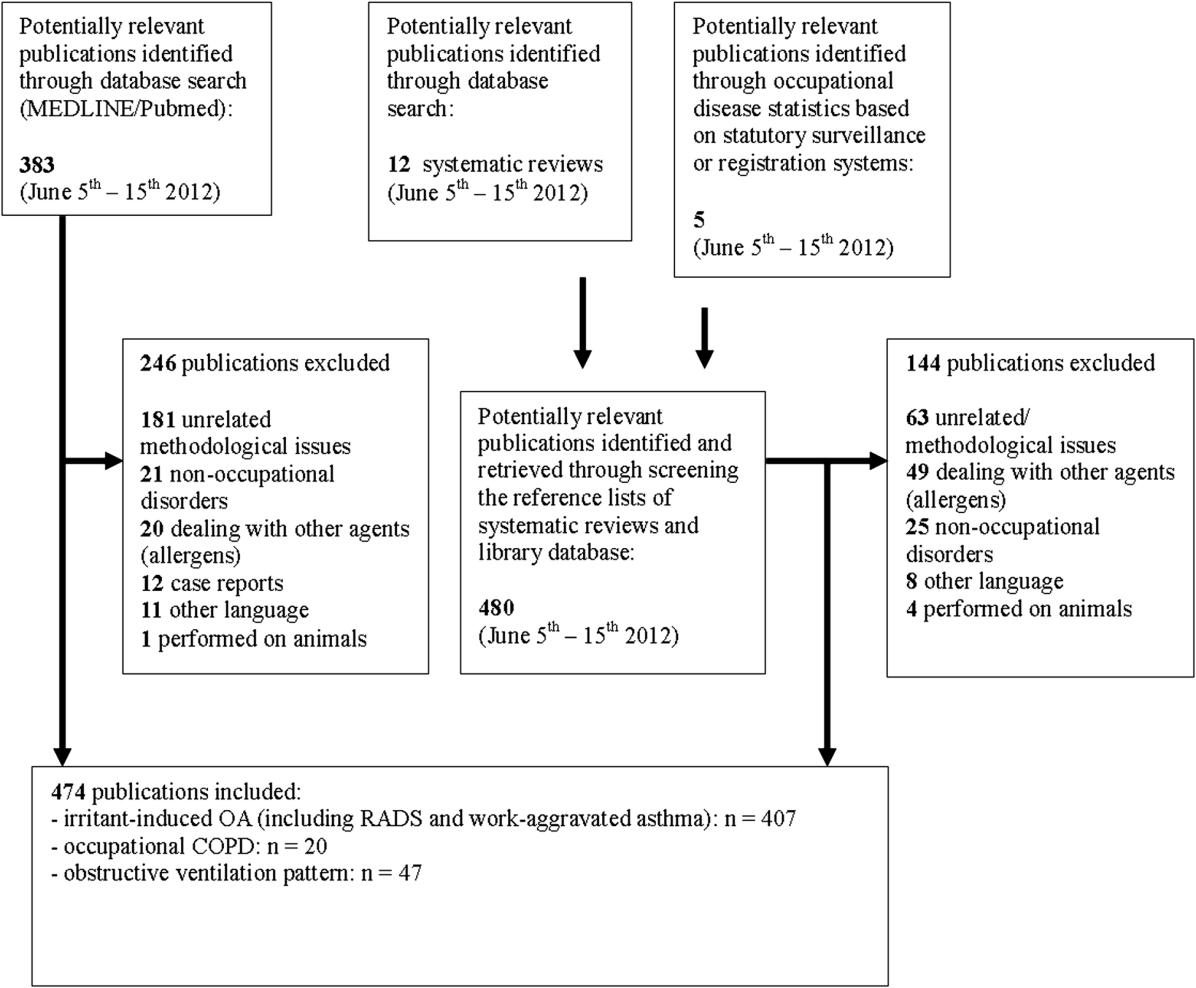
**Flow diagram demonstrating thesource of references.**

The database search (MEDLINE/ PubMed) yielded 383 potentially relevant publications. 480 additional potentially relevant publications were retrieved from the reference lists of 13 systematic reviews or overviews [2,30,36,80,89-97], from occupational diseases routine statistics (SWORD 1994–1997 [47,81-83]; SHIELD 1993 [74]; SORDSA 2001 [84]; SENSOR 2003 [85]; BK-DOK 2007 [86], and from the library of the Institute of Occupational Medicine, Hamburg. Alltogether, the different search approaches yielded a total of 474 relevant studies, including an extreme early study from the year 1932 [98]. (See selection flow diagram, Figure 3).

Most (n = 337) of the 474 relevant publications were identified through hand searching (i.e. reference list checking of systematic reviews and from our library database).

The 474 publications refer to 131 individual agents, 46 to “mixed” agents and 11 to work-sites or professions reported to cause OA and/ or occupational COPD.

### Diagnostic aspects

Many different ways of confirming irritant-induced OA were used, with specific inhalation challenge (SIC) and lung function tests (LFT) being the most reliable diagnostic aids.

SIC was used as the “gold standard” in confirming OA mainly in non-analytical studies (n = 189 studies, i.e. 72% of non-analytical studies). Only few (n = 16, i.e. 7.5% of analytical studies) cohort or case–control reported diagnostic confirmation with SIC.

Another frequently used (n = 191) diagnostic method for OA or occupational COPD was lung function testing (LFT); showing an obstructive ventilation pattern and/or NSBHR related to occupational exposures, mostly in combination with WRA symptoms.

Exclusively self-reported asthma symptoms or physician reported asthma as documented in questionnaires as an alternative diagnosis for OA was used in 36 studies.

Other studies (n = 44) had not clear diagnosis of OA or occupational COPD but reported obstructive ventilation pattern. The number of subjects with asthma symptoms and frequencies of obstructive ventilation patterns and/or NSBHR are provided for each study (see Additional file 3: Table S2E of online supplement “Results”).

### Irritant-induced OA as outcome

Irritant-induced OA was the focus of most studies and was the predominant diagnosis.

RADS, as a subgroup of irritant-induced OA, was reported to be due to 47 different agents, with the most prevalent being the World Trade Center disaster in 2001 (n = 7 studies), chlorine (n = 11), cleaning agents (n = 18) and isocyanates (n = 46). These were followed by disorders, caused by metam sodium (n = 17), ammonia (n = 11), diesel exhaust (n = 10), acids (n = 9), solvents (n = 8), sulfur dioxide (n = 7), dinitrogen tetraoxide (n = 6), hydrogen chloride (n = 4), smoke (fires, pyrolysis products) (n = 4), chlorofluorocarbons (n = 4), spray paint (n = 3), tear gas (n = 3), bromine (n = 2), dichlorvos (n = 2), sodium azide (n = 2), acrylates (n = 1), amprolium hydrochloride (n = 1), phthalic anhydride (n = 1), bromochlorodifluoromethane (n = 1), bromotrifluoromethane (n = 1), chloramine T (n = 1), chromate (n = 1), hydrazine (n = 1), hydrogen fluoride (n = 1), methylmercaptan (n = 1), phosgene (n = 1), uranium hexafluoride (n = 1), airbag content (n = 1), bleaching agent (n = 1), floor sealant (n = 1), fumigant (n = 1), metal coat remover (n = 1), metal oxide fume (n = 1), pesticides (n = 1), refractory ceramic fibers (n = 1), swine confinement (n = 1).

The majority of asthma-inducing agents elicited OA after prolonged exposure and rarely after a single exposure.

Work-aggravated asthma was of less importance in the literature and occurred in only a few studies [30,99-104].

### Occupational COPD as outcome

Ten agents and five professions or work-sites were reported to cause occupational COPD, as shown in Table 2.

**Table 2:** **Agents and professions showingevidence of occupational COPD**

**Agents, number of studies (n)**	**Work-sites or professions, number of studies (n**)
○ ammonia (1) ○ cement dust (4) ○ chlorine (1) ○ cleaning agent (1) ○ mustard gas (1) ○ diesel exhaust (2) ○ environmental tobacco smoke(1) ○ isocyanate (1) ○ smoke (1) ○ sulphur dioxide (1)	○ construction work (3) ○ swine confinement (1) ○ farming (1) ○ foundry (1) ○ metallurgical industry workers (1)

COPD = Chronic obstructive pulmonary disease.

As already mentioned, occupational COPD was not specifically addressed in most of the studies. Some describe respiratory symptoms, such as chronic bronchitis (n = 21), which may be indicative of COPD. One of the few studies which specifically focused on COPD, was a large retrospective cohort study on diesel exhaust which caused a significantly increased COPD mortality in railroad workers after the introduction of diesel engines in 1945 [105,106]. Construction work was identified as a cause of occupational COPD in 2 publications [8,107].

### Evidence level of the literature

Some publications investigated more than one irritant agent and thus have been considered several times in our study.

262 of the 474 publications were non-analytic studies and were rated according to SIGN as 4, 3 or 3+ and consisted of case reports (n = 228), case series (n = 63), and occupational diseases statistics (n = 33) and reviews of that kind of studies (n = 7). The other publications reported analytical studies and were rated according to SIGN as 2+ (n = 15), 2- (n = 103), or 3+ (n = 83).

The highest level was 2+, indicating a well conducted analytical study (case control or cohort studies) with a low risk of confounding or bias (n = 15 studies). Other studies with a similar design had a higher risk of confounding or bias and were individually rated lower by SIGN grading of 2- (n = 30 studies). Most of the other analytical studies were rated with a SIGN grade of 2-, because their design (cross-sectional or longitudinal study) was limiting (n = 82 studies). Cross-sectional studies or longitudinal studies, e.g. those with high risk of confounding or bias, were rated even lower with 3/ 3+ (n = 35 studies). A couple of study designs were difficult to classify epidemiologically, including those which were surveys, mostly with very low analytical evidence, rated 3/ 3+ (n = 53 studies), or larger surveys with a lower risk of confounding or bias, which were graded with 2- (n = 4 studies).

Investigations involving dose–response relationship as a form of scientific evidence were performed in 30 out of 474 studies analyzed [68,105,106,108-133].

Another assessment of the level of evidence found in individual studies is to consider their OR for irritant-induced OA or occupational COPD; this was done in 39 publications [15,23,44,48,105-108,113,117-120,122,126,128,134-156].

### Strength of evidence per agent, work-site or profession

The outcome for each agent causing OA or occupational COPD was graded according to the modified RCGP three-star system to classify the strength of evidence of its causative role in irritant-induced OA/ occupational COPD. The strongest evidence achieved was two stars “**” (indicating a moderate strength of evidence provided by generally consistent findings in fewer, smaller or lower quality scientific studies) for 17 (mixed) agents, work-sites or professions. For six of them (chlorine, platinum salts, environmental tobacco smoke, welding fumes, construction work, World Trade Center disaster in 2001), this level was based on well- conducted studies with low risk of confounding and/or bias (SIGN 2+). For eleven of these 17 (mixed) agents, SIGN levels of individual studies were lower (benzene-1, 2, 4-tricarboxylic acid-1,2-anhydride [trimellitic anhydride], cobalt, isocyanates, cement dust, grain dust, animal farming (pig, beef/veal, dairy, poultry), or swine confinement.

Low to moderate scientific evidence – provided by generally consistent findings in fewer, smaller or lower quality analytical studies, based on questionnaires or other inadequacies, i.e. “*[*]” – was found for 12 agents (phthalic anhydride, glutaraldehyde [glutaral], sulfur dioxide, cotton (dust, raw) CNT 750, potroom aluminum smelting, farming (various) or foundry), smoke (fires, pyrolysis products), pesticides (not specified), cleaning agents (not specified), ceramic production (dust), health care workers.

Limited or contradictory evidence – provided by only one analytical study or inconsistent findings in multiple scientific studies, i.e.“*” – was identified for 39 agents, and after down-grading because of inadequate methodological aspects, i.e. “[*]” on three occasions. For the majority of agents, only non-analytical studies were reported for ≥ 5 cases, i.e. “(*)” or less than 5 cases, i.e. “-”. When only non-analytical studies were available, the strength of evidence for the agent was raised if at least 5 cases were identified by the case reports/ case series or occupational disease statistics for which proof of irritant-induced OA or occupational COPD existed. The strength of evidence reached when only non-analytical studies were available ranged from “very limited or contradictory evidence” in 29 studies, i.e. “(*)”, to “no scientific evidence” “-” 94 times. (see Tables 3 and 4 and Additional file 3: Table S2E of online supplement “Result”).

**Table 3:** **Overview of individual agentscausing irritant-induced OA oroccupational COPD**

**Agent CAS**	**Strength of evidence (modifiedRCGP three star grading)**	**Number of studies peragent**	**References**
**Acids**			
", acetic	*	**3**	[108,157,158]
64-19-7			
", not specified	(*)	**2**	[30,37]
", dodecanedioic	**-**	**1**	[159]
693-23-2-1			
", various	**-**	**1**	[35]
", " (hydrochloric, hydrofluoric, nitric, perchloric, sulfuric)	**-**	**1**	[160]
", hydrochloric	(*)	**6**	[35,99,161,162]
7647-01-0			
", hydrofluoric	**-**	**1**	[163]
7664-39-3			
", sulfuric	*	**3**	[99,109,164]
7664-93-9			
**Acrylates**			
", not specified	**-**	**1**	[165]
", alkyl cyanoacrylates	(*)	**4**	[166-169]
", cyanoacrylate glue	**-**	**1**	[170]
", " [loctite]	*	**4**	[44,169,171,172]
53858-53-0			
", methacrylates	*	**1**	[134]
", methyl 2-cyanoacrylate	**-**	**3**	[166,169,173]
137-05-3			
", methylmethacrylate	**-**	**2**	[169,174]
80-62-6			
**Aluminum salts**	*	**1**	[100]
aluminum fluoride: 7724-18-1			
aluminum sulfate: 10043-01-3			
**2-Aminoethanol [2-ethanolamine]**	**-**	**1**	[175]
141-43-5			
**Amino-ethyl-ethanolamine**	**-**	**1**	[176]
111-41-1			
**3-Amino-5-mercapto-1,2,4-triazole**	(*)	**1**	[177]
16691-43-3			
**Ammonia**	*	**6**	[41,178-182]
7664-41-7			
**Ammonium chloride (triple salt)**	**-**	**1**	[183]
12125-02-9			
**Ammonium thioglycolate**	**-**	**1**	[184]
5421-46-5			
**Amprolium hydrochloride**	**-**	**1**	[185]
137-88-2			
**Anhydrides**			
", various	*	**2**	[186,187]
", dioctyl phthalate	**-**	**1**	[188]
117-81-7			
", hexahydrophthalic	**-**	**1**	[189]
37226-48-5			
", himic	**-**	**1**	[190]
2746-19-2			
", maleic	**-**	**2**	[191,192]
108-31-6			
", methyltetrahydrophthalic	**-**	**1**	[193]
26590-20-9			
", phthalic anhydride	*[*]	**5**	[194-198]
85-44-9			
", pyromellitic dianhydride	**-**	**1**	[199]
89-32-7			
", tetrachlorophthalic anhydride	*	**4**	[200], [201], [202], [203]
117-08-8			
", benzene-1, 2, 4- tricarboxylic acid 1,2-anhydride [trimellitic anhydride]	**	**5**	[197,204-207]
552-30-7			
**Aziridine, polyfunctional**	(*)	**2**	[208,209]
64265-57-2			
**Azobisformamide [azodicarbonamide]**	*	**5**	[210-214]
123-77-3			
**Benzalkonium chloride** (fumes)	**-**	**2**	[215,216]
8001-54-5			
**1, 2-Benzisothiazoline-3-one** (fumes)	**-**	**1**	[217]
2634-33-5			
**Bisulfite*, SO2***	**-**	**1**	[37]
*SO2: 7446-09-5*			
**Bromine, hydrobromic acid**	**-**	**1**	[218]
**Bromochlorodifluoromethane (Halon 1211)**	**-**	**1**	[101]
353-59-3			
**Bromotrifluoromethane (Halon 1301)**	**-**	**1**	[219]
75-63-8			
**Cadmium** (fumes)	*	**4**	[68,220-222]
7440-43-9			
**Calcium carbonate [chalk powder]**	**-**	**1**	[110]
**Calcium oxide**	**-**	**1**	[35]
1305-78-8			
**Captafol** (chlorinated thiocarboximide fungicide)	**-**	**1**	[223]
2425-06-1			
**Carbon black dust**	*	**1**	[224]
1333-86-4			
**Chloramine T** (powder dust)	(*)	**5**	[225-229]
7080-50-4			
**Chlorhexidine**	**-**	**1**	[230]
55-56-1			
**Chlorine**	**	**11**	[35,165,231-239]
7782-50-5			
**Chromate** (not specified)	(*)	**9**	[240,241][98,242-247]
[see also cement]			
**Cobalt**	**	**15**	[74,113,247-259]
7440-48-4			
**3-(Diamino-amino)propylamine** 3-(dimethylamino)propylamine]	(*)	**1**	[260]
109-55-7			
**Diamine, aliphatic + cycloaliphatic** (hardener) 2855-13-2 (isophorone diamine)	**-**	**1**	[261]
**Diazonium tetrafluoroborate** 14239-22-6	**-**	**2**	[262,263]
**Dichlorodiethyl sulfide** [mustard gas] +505-60-2	(*)	**1**	[264]
**Dichlorvos** (organophosphate)	**-**	**2**	[265,266]
62-73-7			
**Diethanolamine**	**-**	**1**	[267]
111-42-2			
**2-Diethylaminoethanol** [diethyl aminoethanol]	(*)	**1**	[268]
100-37-8			
**2-Dimethylaminoethanol** [dimethyl ethanolamine]	**-**	**2**	[269,270]
108-01-0			
**Diinitrogen tetraoxide** [dinitrogentetroxide]	(*)	**1**	[271]
10544-72-6			
**Ethylenediamine** [ethylene diamine]	*	**6**	[168,184,272-275]
107-15-3			
**Ethylene oxide**	**-**	**2**	[276,277]
75-21-8			
**Formaldehyde** (gas, dust)	*	**9**	[278-283], [284,285]
50-00-0			
**Freon**, (freon-22)	**-**	**2**	[286,287]
**Glutaraldehyde** [glutaral]	*[*]	**9**	[74,83,288-294]
11-30-8			
**Hexachlorophene**	**-**	**1**	[295]
70-30-4			
**Hexamethylenetetramine**	*	**3**	[184,296,297]
100-97-0			
**Hydrazine**	**-**	**1**	[30]
302-01-2			
**Iridium salt**	**-**	**1**	[298]
**Isocyanates, isocyanurate**			
", various (HDI, MDI, TDI)	**	**11**	[57,83,148,149,165,281,299-303]
", Diphenylmethane diisocyanate [MDI]	**	**7**	[304-310]
5873-54-1			
", " prepolymers	**-**	**1**	[311]
", Hexamethylene diisocyanate [HDI]; plus isodurane Diisocyanate	(*)	**3**	[312,313]
822-06-0			
", HDI biuret plus			[314]
4035-89-6			
", 3-Isocyanatomethyl-3,5,5-trimethylcyclohexyl isocyanate [isophorone diisocyanate, IPDI]	**-**	**1**	[315]
4098-71-9			
", Methyl isocyanate [MIC]	*	**6**	[316-321]
624-83-9			
", 1,5-Naphthylene diisocyanate [NDI]	(*)	[46]	[322-324]
3173-72-6			
", Polymethylene polyphenyl isocyanate	*	**1**	[325]
9016-87-9			
", Toluene diisocyanate, TDI 2,4: 584-84-9;	**	**12**	[35,125,165,326-334]
2,6:91-08-7			
", Triglycidil isocyanurate	**-**	**1**	[335]
2451-62-1			
", Triphenylmethane triisocyanate	**-**	**1**	[336]
**Isothiazolinone**	**-**	**1**	[337]
55965-84-9			
**Lauryl dimethyl benzyl ammoniumchloride**	**-**	**1**	[338]
139-07-1			
**Metam sodium** [methyldithiocarbamate]	**-**	**1**	[102]
144-54-7			
**Methylmercaptan**	**-**	**1**	[165]
74-93-1			
**Monoethanolamine**	**-**	**1**	[184]
141-43-5			
**N-methylmorpholine**	[*]	**1**	[339]
109-02-4			
**Nickel sulphate**	(*)	**5**	[246,340-343]
→anhydrous 7786-81-4			
→hexahydrate 10101-97-0			
**Ninhydrin** 485-47-2	**-**	**1**	[335]
**Nitrogen chloride** [nitrogen trichloride, trichloramine]	[*]	**2**	[150,344]
10025-85-1			
**Ozone** (gassings)	*	**1**	[345]
10028-15-6			
**Palladium**	**-**	**1**	[346]
7440-05-3			
**Paraphenylenediamine**	(*)	**1**	[347]
106-50-3			
**Paraquat**	*	**2**	[128,151]
4685-14-7			
**Persulfate**			
", not specified	(*)	**2**	[348,349]
", ammonium	**-**	**1**	[350]
", potassium (7727-21-1) and ammonium peroxydisufate	[*]	**5**	[351]
(7727-54-0)			
", alcalic	**-**	**1**	[352]
", Sodium persulfate	**-**	**1**	[353]
7775-27-1			
", Dipotassium peroxo-peroxodisulfate [potassium persulfate] 7727-21-1	**-**	**1**	[354]
", Diammonium peroxodisulfate [ammonium persulfate]	*	**4**	[355-357]
7727-54-0			
**Phenylglycine acid chloride**	*	**1**	[358]
39478-47-2			
**Phosgene**	**-**	**2**	[35,359]
75-44-5			
**Piperazine dihydrochloride**	*	**3**	[130,176,274]
142-64-3			
**Platinum salts**	**	**8**	[131,176,360-365]
(7440-06-4)			
**Polyethylene**	**-**	**3**	[366-368]
9002-88-4			
**Polymethyl-methacrylate** [plexiglas powder]	**-**	**1**	[369]
9011-14-7			
**Polypropylene**, heated to 250 °C	[*]	**2**	[370,371]
9003-07-0			
**Polyvinyl chloride** (fume)	*	**8**	[53,372-376][377,378]
9002-86-2			
**Potassium dichromate**	(*)	**1**	[379]
7778-50-9 ( see also chromium;cement)			
**Potassium aluminum tetrafluoride**	(*)	**1**	[380]
14484-69-6			
**Rosin core solder**, thermal decomposition [colophony]	*	**6**	[74,83,381-383]
8050-09-7			
**Sodium azide** (powder dust)	**-**	**1**	[384]
26628-22-8			
**Sodium iso-nonanoyl oxybenzene sulphonate** [SINOS] 123354-92-7	(*)	**3**	[385-387]
**Sodium metabisulfite** [metabisulfite sodium]	(*)	**6**	[103,388-392]
7681-57-4			
**Styrene monomer**	(*)	**3**	[132,393,394]
100-42-5			
**Sulfur dioxide**	*[*]	**5**	[35,154,395-397]
7446-09-5			
**Sulfathiazole**	**-**	**1**	[398]
72-14-0			
**Terpene** (3-carene)	**-**	**2**	[399,400]
13466-78-9			
**Tetrachloroisophthalonitrile** (fungicide)	**-**	**1**	[401]
**Tetrahydrothiophene**	**-**	**1**	[402]
110-01-0			
**Tetramethrin** [1-(5-tretrazoly)- 4-guanyl-tetrazene hydrate] 7696-12-0	**-**	**2**	[338,403]
**Tributyl tin oxide** [carpet fungicide]	**-**	**1**	[404]
**Triethanolamine**	**-**	**1**	[175]
102-71-6			
**Tungsten carbide**	**-**	**1**	[405]
11130-73-7			
**Tylosin tartrate**	**-**	**1**	[406]
**Uranium hexafluoride**	**-**	**2**	[30,407]
7783-81-5			
**Urea** (fume)	**-**	**1**	[104]
57-13-6			
**Urea formaldehyde foam**	**-**	**1**	[408]
64869-57-4**/**			
**Phenol-formaldehyde resin**			
9003-35-4			
**Vanadium** 7440-62-2 **+ divanadium pentoxide**	*	**5**	[84,409-412]
1314-62-1			
**Zinc** (fume)	**-**	**3**	[413-415]
7440-66-6			
**Zinc chloride** (fume)	**-**	**1**	[183]
7646-85-7			
**Mixed agent**	**Evidence level**	**Number of studies peragent**	
**Acid fluxes**	**-**	**1**	[74]
**Acrylic acid**	**-**	**1**	[166]
**Airbag content**	**-**	**1**	[416]
**Bleaching agent (fumes)**	**-**	**1**	[99]
**Cement**	**	**14**	[111,133,235,417-427]
65997-15-1			
**Chlorofluorocarbons**	(*)	**2**	[428,429]
(degradation products)			
**Cleaning agents** (not specified)	*[*]	**9**	[15,37,41,112,135,430-433]
", detergents	**-**	**2**	[434,435]
**Coffee, green**	*	**5**	[436-440]
**Cotton** (dust, raw)	*[*]	**(12)**	[48,114-116,423,441-447]
CNT 750			
**Cutting oil**	**-**	**2**	[37,448]
**Diesel exhaust**	*	**5**	[10,45,46,105,106]
**ECG ink**	**-**	**2**	[449,450]
**Endotoxin** (see also cotton dust, swine confinement, poultry confinement, house dust)	*	**2**	[41,451]
**Environmental tobacco smoke**	**	**10**	[117,118,138-143,452,453]
**Floor sealant** (aromatic hydrocarbons)	**-**	**1**	[30]
**Fumigating agent**	**-**	**1**	[30]
**Furan-based binder**	**-**	**1**	[454]
**Grain**	**	**9**	[48,122-124,455-459]
**", rice**	[*]	**1**	[460]
**Hairdressing chemicals**	(*)	**1**	[281]
**Lubricants** (not specified)	(*)	**2**	[10,57]
**Metal coat remover** (coating removing chemical)	**-**	**1**	[30]
**Metal oxide** (fume)	**-**	**1**	[461]
**Metal working fluids** [MWF]	**-**	**1**	[462]
**Oil** (spill)	*	**1**	[126]
**Paint** (fumes)	*	**4**	[35,127,463,464]
**Paper dust A111**	(*)	**1**	[48]
**Perfume agents** (research lab)	**-**	**1**	[37,465]
**Pesticides** (not specified)	*[*]	**5**	[129,152,266,466,467]
**Polyamines**, aliphatic	[*]	**1**	[468]
**Polyester**	(*)	**2**	[469,470]
**Potroom aluminum smelting**	*[*]	**10**	[52,247,471-478]
**Powder paints**	(*)	**1**	[479]
**Pyrazolone** (see reactive dye)	**-**	**1**	[480]
**Reactive dyes**	*	**5**	[481-485]
**Refractory ceramic fibers** [RCF]	*	**2**	[486,487]
**Smoke (fires, pyrolysis products)**	*[*]	**5**	[30,41,488-490]
", **(oil fire and duststorm)**	*	**1**	[491]
", **(biomass, indoor)**	(*)	**1**	[492]
**Soldering flux** (fumes)	*	**4**	[183,493-495]
**Solvents** (not specified)	*	**4**	[10,48,496,497]
**Spray paint**	**-**	**1**	[30]
**Tall oil**	**-**	**1**	[498]
**Tear gas**	**-**	**4**	[499-502]
**Welding fumes**	**	**18**	[41,48,57,83,247,281,464,503-513]
**Work-site or profession**	**Evidence level**	**Number of studies perwork-site or profession**	
**Ceramic production**	*[*]	**2**	[514,515]
**Cleaners**			
**Construction work** (dust, agent not specified)	**	**5**	[8,107,136,516,517]
**Farming**			
", (various)	*[*]	**9**	[119,120,144-146,518-521]
", animals (pig, beef/veal, dairy, poultry)	**	**3**	[522-524]
**Foundry**	*[*]	**4**	[121,147,525,526]
[see also isocyanates (MDI)]			
**Health care workers**	*[*]	**4**	[23,527-529]
**Metallurgical industry workers**	*	**1**	[530]
**Poultry confinement**	*(*)	**4**	[153,531-533]
**Poultry confinement, slaughtery house**	*	**3**	[534-536]
**Swine confinement**	**	**8**	[39,40,141,155,537-540]
**World Trade Center disaster2001**	**	**8**	[133,156,541-546]

[ ] down-grading due to lower quality of clinical investigations relative to the scale of the scientific level of the study.

(*) up-grading due to at least 5 cases without contradictory findings.

**Table 4:** **Strength of evidence foragents, professions and work-siteaccording to the modifiedRCGP three-star system**[88]

**Evidence level (modified RCGPthree-star grading)**	**Number of agents/work-sites orprofessions**	**Agents, work-site or profession [Synonym] (CAS)**
***	0	-
**	17	Benzene-1, 2, 4-tricarboxylic acid-1,2-anhydride [trimellitic anhydride] (552-30-7); chlorine **(**7782-50-5); cobalt (7440-48-4); various isocyanates, isocyanurate (HDI, MDI, TDI), diphenylmethane diisocyanate [MDI] (5873-54-1), toluene diisocyanate, TDI 2,4 (584-84-9), TDI 2,6: (91-08-7); platinum salts (7440-06-4); cement ; environmental tobacco smoke; grain ; welding fumes; construction work (dust, agent not specified); farming, animals (pig, beef/veal, dairy, poultry); swine confinement; World Trade Center disaster 2001
*[*]	12	Ceramic production; Phthalic anhydride (85-44-9); glutaraldehyde [glutaral] (11-30-8); sulfur dioxide (7446-09-5); cotton (dust, raw) CNT 750; potroom aluminum smelting; farming (various); foundry; smoke (fires, pyrolysis products); pesticides (not specified); cleaning agents (not specified); health care workers
*	39	Acetic acid (64-19-7); sulfuric acid (7664-93-9); metacrylates, loctide® (53858-53-0); aluminum salts [aluminum fluoride] (7724-18-1); aluminum sulfate: (10043-01-3); ammonia (7664-41-7); various anhydrides; tetrachlorophthalic anhydride (117-08-8); azobisformamide (123-77-3); cadmium (fumes) (7440-43-9); carbon black dust (1333-86-4); ethylenediamine (107-15-3); formaldehyde (gas, dust) (50-00-0); hexamethylenetetramine **(**100-97-0); methyl isocyanate [MIC] (624-83-9); naphthylene diisocyanate (3173-72-6); polymethylene polyphenyl isocyanate (9016-87-9); N-methylmorpholine (09-02-4); ozone (gassings) (10028-15-6); paraquat (4685-14-7); diammonium peroxodisulfate (7727-54-0); phenylglycine acid chloride **(**39478-47-2); piperazine dihydrochloride (142-64-3); polyvinyl chloride (fume) (9002-86-2); rosin core solder; thermal decomposition (8050-09-7); vanadium (7440-62-2) + divanadium pentoxide (1314-62-1); cleaning agents (not specified); green coffee ; diesel exhaust; endotoxin; oil (spill); paint (fumes); pesticides (not specified); reactive dyes; refractory ceramic fibers [RCF]; smoke (fires, pyrolysis products; oil fire and dust storm); soldering flux; solvents (not specified); health care workers; poultry confinement; slaughtery house; metallurgical industry workers
[*]	3	Nitrogen chloride **(**10025-85-1); polyamines, aliphatic; potassium persulfate (7727-21-1) and ammonium peroxydisufate (7727-54-0); grain rice
(*)	29	Acids not specified; hydrochloric acids (7647-01-0); alkyl cyanoacrylates; 3-amino-5-mercapto-1,2,4-triazole l(16691-43-3); aziridine, polyfunctional (64265-57-2); chloramine T (powder dust) (7080-50-4); chromate (not specified); 3-(diamino-amino)propylamine (109-55-7); dichlorodiethyl sulfide (505-60-2); 2-diethylaminoethanol (100-37-8); diinitrogen tetraoxide (10544-72-6); hexamethylene diisocyanate [HDI], plus isodurane diisocyanate (822-06-0); HDI biuret plus (4035-89-6); nickel sulphate anhydrous (7786-81-4); hexahydrate (10101-97-0); paraphenylenediamine (106-50-3); persulfate (not specified); polypropylene, heated to 250 °C (9003-07-0); potassium dichromate (7778-50-9); potassium aluminum tetrafluoride (14484-69-6); sodium iso-nonanoyl oxybenzene sulphonate [SINOS] (123354-92-7); sodium metabisulfite (7681-57-4); styrene monomer (100-42-5); chlorofluorocarbons (degradation products); hairdressing chemicals; lubricants (not specified); paper dust A111; aliphatic polyamines; polyester; powder paints; smoke (biomass, indoor)
**-**	93	Acids various; dodecanedioic (693-23-2-1); hydrofluoric acids (7664-39-3); cyanoacrylate glue; methyl 2-cyanoacrylate (137-05-3); methylmethacrylate (80-62-6); 2-aminoethanol (141-43-5); amino-ethyl-ethanolamine (111-41-1); ammonium chloride (triple salt) (12125-02-9); ammonium thioglycolate (5421-46-5); amprolium hydrochloride (137-88-2); dioctyl phthalate (117-81-7); hexahydrophthalic anhydrides (37226-48-5); himic anhydrides (2746-19-2); maleic anhydrides (108-31-6); methyltetrahydrophthalic anhydrides (26590-20-9); pyromellitic dianhydride (89-32-7); benzalkonium chloride (fumes) (8001-54-5); 1, 2-benzisothiazoline-3-one (fumes) (2634-33-5); bisulfite*, SO2*:(7446-09-5*);* hydrobromic acid bromine; bromochlorodifluoromethane [halon 1211] (353-59-3); bromotrifluoromethane [halon 1301](75-63-8); calcium carbonate [chalk powder]; calcium oxide (1305-78-8); captafol (2425-06-1); chlorhexidine (55-56-1); aliphatic + cycloaliphatic diamine, (hardener) (2855-13-2) [isophorone diamine]; diazonium tetrafluoroborate (14239-22-6); dichlorvos [organophosphate] (62-73-7); diethanolamine (111-42-2); 2-dimethylaminoethanol [dimethyl ethanolamine] (108-01-0); ethylene oxide (75-21-8); freon-22; hexachlorophene (70-30-4); hydrazine (302-01-2); iridium salt; isocyanate prepolymers; 3-isocyanatomethyl-3,5,5-trimethylcyclohexyl isocyanate (4098-71-9); triglycidil isocyanurate (2451-62-1); triphenylmethane triisocyanate; isothiazolinone (55965-84-9); lauryl dimethyl benzyl ammonium chloride (139-07-1); metam sodium (144-54-7); methylmercaptan (74-93-1); monoethanolamine (141-43-5); ninhydrin (485-47-2); palladium (7440-05-3); ammonium persulfate; alcalic persulfate; sodium persulfate (7775-27-1); dipotassium peroxo-peroxodisulfate (7727-21-1); phosgene (75-44-5); polyethylene (9002-88-4); polymethyl-methacrylate (9011-14-7); sodium azide (powder dust) (26628-22-8); sulfathiazole (2-14-0); terpene (3-carene) (3466-78-9); tetrahydrothiophene (110-01-0); tetrachloroisophthalonitrile (fungicide); tetramethrin (7696-12-0); tributyl tin oxide; triethanolamine (102-71-6); tungsten carbide (11130-73-7); turpentine (8006-64-2); tylosin tartrate; uranium hexafluoride (7783-81-5); urea (fume) (57-13-6); urea formaldehyde foam (64869-57-4); phenol-formaldehyde resin (9003-35-4); zinc (fume) (7440-66-6); zinc chloride (fume) (7646-85-7); acid fluxes; acrylic acid; airbag content; bleaching agent (fumes); chlorofluorocarbons (degradation products); detergents; cutting oil; ECG ink; floor sealant (aromatic hydrocarbons); fumigating agent; furan-based binder; metal coating remover (coating removing chemical); metal oxide (fume); metal working fluids; perfume agents (research lab); pyrazolone; spray paint; tall oil; tear gas

CAS = Chemical abstracts service.

RCGP = Royal College of General Practitioners.

The compiled assessment of the individual studies, along with their relevant clinical data and strength of evidence for irritant agents, professions or workplaces causing asthma or COPD, is presented as a summary list (see Additional file 3: Table S2E “Results” for the full information).

## Discussion

The main objective of this study was to give a comprehensive and evidence-based overview of the literature on irritative agents, professions or work-sites causing irritant-induced work-related asthma and occupational COPD. To our knowledge this study is the first attempts to document these respiratory disorders, along with their causative irritant agents in an evidence-based manner.

The 474 publications retrieved (see Table 3 and Additional file 3: Table S2E of online supplement “Results”) in this work mainly refer to individual agents (n = 131), but also to mixed exposure(s) or multicomponent work-sites or professions (n = 57) where heterogeneous exposure to irritating substances is common, e.g. swine confinement, “construction work” or “farming”, giving 188 different causes of irritant-induced OA and/or occupational COPD in total.

### Strength and limitations

This work covers a broad range of causative agents of irritant-induced occupational asthma or COPD. We included various study designs.

A strength of our work is that we not only assessed the quality of single investigations but the strength of the body of evidence for each irritant agent.

The paradigm of “evidenced-based medicine” has been criticized by leading scientists [547-550]. Bias in the selection of information may be a problem for generalization of findings in single studies [551,552]. In spite of these limitations, alternative approaches to evaluation of the literature have not been generally accepted. Evaluation of the evidence depends on the domain, which means the factors to be considered in assessing the extent to which the study results are reliable or valid.

Kunz et al. [553] stressed the approach of grading scientific studies on basis of additional qualified data, i.e. dose response relationships. This latter was seen in 30/474 individual studies in this current work. Other studies were based on evidence by OR >2 or < 0.5 for irritant-induced OA and occupational COPD which was applied as an approach in 40/474 individual studies (see Additional file 3: Table S2E of online supplement “Results”).

There are numerous procedural methods for rating the strength of scientific evidence. The AHRQ emphasized in 2002: “systems for grading the strength of a body of evidence are much less uniform than those for rating study quality” [554].

It is possible that not all relevant studies were found in our search of literature. Probably, some studies could not be found by the MeSH term raster applied. Relying solely on MeSH terms might be a problem in the identification of studies of irritant-induced OA or occupational COPD. We restricted the search to the MeSH fields in order to increase the specificity of the search. As for any electronic search strategy, an increase of specificity implies a decrease in sensitivity of the search.

For each single study, we took into consideration possible risks due to confounding, e.g. exposure to multiple agents and selection bias, e.g. healthy worker effect.

### Basis and quality of data

Irritant-induced obstructive airways diseases cannot usually be diagnosed in one clinical visit and, instead, follow-up and/or detailed clinical investigations are necessary. The diagnostic “gold standard” for OA is SIC using a specific occupational agent in an exposure chamber. SIC is particularly indicated in the clinical setting where new causative substances with still unknown adverse respiratory sensitization potential are suspected. This “gold standard” is not applicable for large studies; so, it was used mainly in case series or reports.The evidence levels to confirm irritant-induced work-relaated asthma or occupational COPD for the listed irritant agents, professions or worksites (see Additional file 3: Table S2E of online supplement “Results”) are frequently low with the major reasons being that high quality studies were missing and the quality of the available studies was low. Nevertheless, this knowledge is the best available and may help physicians to identify a suspected irritant agent as causative in irritant-induced work-related asthma and / or occupational COPD [555]. As also recently stressed by Quint et al. [555], “implementing an evidence-based identification and regulatory process for OA will help to ensure primary prevention of OA”. In cases of low evidence level of an agent that does not exclude a causative role, caution should be exercised and a more detailed diagnostic testing of relevant exposure should be performed.

### Occupational COPD, an underestimated category

We identified only 20 out of 474 publications that referred to occupational COPD, with most of them implicating inorganic or organic dust or fumes, such as cement dust, construction work and diesel exhaust, as the causative agents.

As an example, the mixed agent cement dust was investigated in 14 studies but only four studies documented cement dust as the causative agent in occupational COPD [111,418,419,422] (see Table 3 and Additional file 3: Table S2E “Results”). The remaining 10 studies described irritant-induced OA cases [235,423-426,530] or identified significant asthma symptoms/ obstructive ventilation patterns without a clear diagnosis (5 studies: [178,417,420,556]). It can be assumed that if it had been considered on the other 10 studies then occupational COPD caused by cement dust would have been frequently observed.

The population-attributable fraction for COPD associated with occupational exposure has been estimated between 9% and 31% [1,64,65]. However the true population-attributable risk due to occupational exposure is unclear [6,557] as occupational COPD is rarely clinically diagnosed. Blanc et al. [558] recently published an ecological analysis using data from three large studies, comprising the Burden of Obstructive Lung Disease study [169], the Latin American Project for Investigation of Obstructive Lung Disease (PLATINO) and the European Community Respiratory Health Survey follow-up (ECHRS II), where occupational COPD was also not a primary goal. The original publications are mainly concerned with OA or asthma symptoms, but a history of pre-existing OA or RADS cannot be allowed to exclude occupational COPD [559]. Blanc et al. [558] stressed that the contribution of occupational exposure cannot be ignored, because “the association between adverse working conditions and COPD (…) carries significance as a global finding (…), alongside the (…) critical contribution of cigarette smoking to disease prevalence”.

General acceptance of this statement does not exist [66,559], although evidence for an association between individual exposure levels and COPD is accumulating in the latest literature [1,6,106,506,557,560,561].

### Irritant-induced WRA – a broader definition

Irritant-induced OA includes three subcategories that predominantly differ according to the concentration of irritants in the workplace atmosphere. It can occur without a latency period, such as RADS, as was shown for 46 causative agents in our study, with the highest prevalence after spills of acids or tear gas (see Additional file 3: Table S2E of online supplement“Results”). Other agents, e.g. isocyanates or welding fumes, usually induce a slower onset of low dose irritant-induced asthma with a latency period and mostly without evidence of an IgE-mediated pathomechanism.

The ACCP also stated in its last Consensus Statement in 2008 [24] that cases who do not meet the stringent criteria of RADS [30] (e.g. where there is a lag of several days before the onset of symptoms or where there is no single massive exposure but rather repeated exposure over days and weeks) should be subsumed into a broader category of irritant-induced OA. As outlined in the section “Introduction” Brooks et al. [31] and later also others, e.g. Burge [27] suggested using the term “not so sudden onset of irritant-induced asthma” for those developing the disorder after such exposure within a period of 2 days to 4 months. In an extended definition corresponding to ours, Burge [27] he used the term “low dose irritant-induced OA” for those developing the disorder after relatively low repeated exposure for more than 4 months.

Bardana [562] and Vandenplas and Malo [563] questioned whether such rather low concentrations could actually cause irritant-induced OA. These different opinions about the pathogenetic role of chronic or recurrent exposure(s) to low concentrations of respiratory irritants seem to be due to inadequate considering of the increased susceptibility of a small group of workers. Occupational disease statistics do mostly neither contain such cases nor work-aggravated asthma cases so far.

Another critical issue is the frequent disregarding of work-aggravated asthma due to occupational agents by physicians.

### Comparison to occupational guidelines or consensus statements – what is new?

In the current analysis, the focus has been on irritant agents causing irritant-induced occupational asthma and COPD. Both entities have been underestimated or even overlooked in the past. Occupational COPD has not been considered as a subgroup of COPD thus so far [559,564]; and the definition of irritant-induced OA has been heterogenous at best [24,552,565,566]. Furthermore, the guidelines dealing with respiratory disorders have not even considered causation by individual irritant agents, so far.

The ACCP published a Consensus Statement in 2008 [24] which focuses on the diagnosis and management of WRA after a latency period, i.e. due allergens and “sensitizers” with unknown pathomechanisms, effectively sidelining irritant-induced OA to RADS.

The Agency for Healthcare Research and Quality (AHRQ) in its the Evidence Report “Diagnosis and Management of WRA” [552] addressed the key question of the best diagnostic approach for a patient with suspected WRA. In respect of irritant-induced OA, they only considered RADS as a non-allergic asthma due to mainly low molecular weight compounds of unknown pathomechanism.

The Canadian Thoracic Society “Guidelines for OA” [567] was the first evidence-based guideline, although irritant-induced OA was limited to RADS. If criteria were not fulfilled then irritant-induced OA was discussed as a controversial diagnosis. The three evidence levels in the “Guidelines for OA” were based on quality of scientific evidence within analyzed studies [568]. Compared with the modified RCGP three-star grading (see Additional file 2: Table S2D in online supplement “Methodology”), the different levels are defined in a more general way, i.e. not considering the quantitative aspect if only studies with lower scientific evidence exist.

The evidence review and recommendations for OA by the BOHRF [3,569] were designed to improve the prevention, identification and management of OA. This work mainly deals with asthma after a latency period and considers irritant-induced OA and RADS to be closely related entities. The difference in comparison with our analysis is obvious even though our evidence-based approach was closely related to the BOHRF guidelines and used the same grading systems.

In summary, the existing guidelines or statements mostly define irritant-induced OA as RADS. Work-aggravated asthma, and occupational COPD as a distinct entity, have not been considered in any guideline, although the latter is becoming recognized as such in more recent publications [557,559,564].

This evidence-based approach is the first which focuses on especially irritative agents within the broader definition of irritant-induced OA and occupational COPD. For clarification, the grading systems were modified in accordance with BOHRF [3] when considering the extent and quality of the clinical investigations, with the goal of creating evidence levels for causative irritative agents as precisely as possible.

## Concluding remarks

OA is the most common chronic occupational lung disease in many industrialized countries [3]. COPD is the fourth leading cause of death worldwide with a significant portion of occupational cases [66]. The term occupational COPD does not officially exist. However, it has to be considered as a subcategory of COPD [559].

Our study shows that reliable, sensitive and specific methods are required in the diagnostic approach for confirming irritant-induced OA, work-aggravated asthma, or occupational COPD. The specific diagnostic work-up in a subject with such a suspected disorder depends on the individual clinical data and on the knowledge of asthma- or COPD-inducing agents in the workplace. On this basis, our review may help in diagnostics especially for agent exposures where we were able to relate irritant-induced work-related asthma or occupational COPD to a high evidence-based level (i.e. two stars according to the RCGP grading).

We have created a list representing the strength of evidence for irritating agents to be causative in irritant-induced work-related asthma or occupational COPD (see Additional file 3: Table S2E of online supplement “Results”).

A low level or absence of evidence for many agents in causing irritant-induced work-related asthma or occupational COPD is sometimes due to contradictory findings in literature, but is mostly due to the absence of rigorous scientific studies, with many gaps remaining in the knowledge of a causative role for individual agents and conditions. Therefore, and because of rarely applied diagnostic approach in the clinical setting, our literature search and evaluation lead us to assume that irritant-induced respiratory disorders are considerably underreported in cross-sectional studies and occupational disease statistics.

Our list needs updating in the light of recent literature, in order to provide a realistic overview of agents and evidence level in their causation of irritant-induced work-related or occupational COPD.

The estimated high population-attributable risk in the range of 5–25% for occupational asthma and COPD from occupational exposure, indicates that more detailed and intensive research, as well as strategies designed to prevent these disorders, should receive high priority in the global efforts to reduce the burden of these diseases. This implies extended evidence-based diagnostic procedures that help to optimize primary and secondary prevention by the physicians dealing with occupational diseases.

Reduction of the exposure to noxious agents by lowering the permissible exposure limits is the best and favoured way for intervention. If this is not possible then other effective primary preventive measures, such as wearing adequate respiratory devices, are required [28,570-574].

Finally, we would like to mention that the diagnosis of irritant-induced OA should be considered if:

there has been exposure to high concentration of an irritative agent identified in this study and the development of asthma without a latency period (original definition of RADS) or

there has been chronic or repeated exposures to moderate (in the TLV ranges) concentrations of an irritative agent identified in this review and the development of asthma with a latency period, but without evidence of an IgE-mediated pathomechanism and

there is evidence that a highly susceptible subject (e.g. with pre-existing NSBHR) develops new onset asthma upon occupational exposure to an identified irritative agent even at concentrations below the TLV.

Work-aggravated asthma should be considered if:

there have been any of the before-mentioned exposures and

there is a temporally related significant worsening of a pre-existing asthma or of a concomitant non-occupational asthma.

The diagnosis of occupational COPD should be considered if:

there has been exposure to an agent capable of causing occupational COPD, and

not reversible chronic airway disease is demonstrated and

there is a temporal relationship between the period of exposure (mostly cumulative exposures to identified irritants ) and the development of COPD (acute WRA symptoms are frequently missing).

Occupational COPD has to be taken into consideration especially in non-smokers, i.e. when dominating non-occupational causes for COPD are obviously not present.

## Endnotes

^a^Epidemiologic study design which is generally applied to test one or more specific hypotheses, typically whether an exposure is a risk factor for a disease [575].

## Abbreviations

CAS: Chemical abstracts service; COPD: Chronic obstructive pulmonary disease; OA: Occupational asthma; RADS: Reactive airways dysfunction syndrome; RCGP: Royal college of general practitioners; SIGN: Scottish intercollegiate guideline network; WRA: Work-related asthma.

## Competing interests

The authors declare that they have no conflict of interest.

## Authors' contributions

All authors made substantial contributions to the study. XB made the design of the study and the final interpretation of data. HV and PB did the detailed literature search, data extraction and analyses, and statistical analyses. XB and HV wrote the manuscript with input from PB. All authors approved the final version for submission.

## Supplementary Material

Additional file 1 Economic burden. Click here for file

Additional file 2 **“Methodology” Selection criteria, information sources, strength of evidence.** Table A: Data extraction and synthesis. Table B. Quality assessment of individual study. Table C - The revised Scottish Intercollegiate Guidelines Network (SIGN) grading system (modifications are given in italics) [87]. Table D The Royal College of General Practitioners (RCGP) three-star system [88] used by the British Occupational Health Research Foundation [3,574] (modifications are given in italics). Click here for file

Additional file 3 **“Results” Table E overview on publications and SIGN grading of reporting OA or occupational COPD due to irritants.** X. Baur, P. Bakehe, H. Vellguth http://www.eomsociety.org = > Knowledge Center. Click here for file
