# Supplementary material for: Bronchial asthma and COPD due to irritants in the workplace - an evidence-based approach
Source: J Occup Med Toxicol. 2012 Sep 26;7:19. doi: 10.1186/1745-6673-7-19 (PMC3508803; doi:10.1186/1745-6673-7-19)
Supplement: Additional file 1 — Economic burden. [file 1745-6673-7-19-S1.docx]

**Online Supplement “Economic Burden”**

**Economic burden**

Based on estimations of new OA cases in Great Britain in 2003 (n= 631), the total lifetime costs to the British society are in the range of €83,1 to €114.8 million or about €3.9 to €5.5 million per year over a lifetime span [[1](#_ENREF_1)]. Estimated medical costs for OA in the United States were $1.5 billion in 1999 [[2](#_ENREF_2)], cited in Leigh et al. [[3](#_ENREF_3)]), and $6.6 billion for combined cost of OA and occupational COPD in 1996 [[4](#_ENREF_4)].

In the year of 2009, Germany’s financial burden from occupational disease claims for “Obstructive respiratory tract diseases caused by chemical irritants or substances with toxic effect”, which is officially listed as occupational disease no. 4302 (German Occupational Disease Act) [[5](#_ENREF_5)], was about € 32 million. This amount of money included all expenditures related to that disease, i.e. compensation for confirmed cases, additionally payments for retraining costs of preventive interventions to avoid the onset of this occupational disease [[6](#_ENREF_6)]. In 2009, 1,437 possible cases referring to the occupational disease no. 4302 were officially reported to the accident insurance institutions. Less than 10% (n= 128) were acknowledged as new cases of the occupational disease no. 4302 .[[7](#_ENREF_7)]

In order to reduce the enormous social burden a significant decline of the number of WRA cases must be realized, through the improvement of effective preventive measures. This requires detailed knowledge on causative conditions, however, which remain unclear, given the current lack of evidence-based identification of WRA inducing agents [[2](#_ENREF_2)].

1. Boyd R, Cowie H, Hurley F, Ayres J: **The true cost of occupational asthma in Great Britain.** Suffolk: Health & Safety Executive (HSE); 2006.

2. Quint J, Beckett WS, Campleman SL, Sutton P, Prudhomme J, Flattery J, Harrison R, Cowan B, Kreutzer R: **Primary prevention of occupational asthma: identifying and controlling exposures to asthma-causing agents.** *Am J Ind Med* 2008, **51:**477-491.

3. Leigh JP, Yasmeen S, Miller TR: **Medical costs of fourteen occupational illnesses in the United States in 1999.** *Scand J Work Environ Health* 2003, **29:**304-313.

4. Leigh JP, Romano PS, Schenker MB, Kreiss K: **Costs of occupational COPD and asthma.** *Chest* 2002, **121:**264-272.

5. Haupt B, Drechsel-Schlund C, Guldner K, Rogosky E, Plinske W, Butz M: *Dokumentation des Berufskrankheiten-Geschehens in Deutschland. BK-DOK 2005.* Sankt Augustin. <http://www.dguv.de/inhalt/zahlen/documents/bk_dok_2005.pdf:> Deutsche Gesetzliche Unfallversicherung (DGUV); 2007.

6. Deutsche Gesetzliche Unfallversicherung (DGUV): **BK 4302, Kosten.** Sankt Augustin: DGUV, Referat BK-Statistik/ZIGUV; 2010.

7. Deutsche Gesetzliche Unfallversicherung (DGUV) (Ed.). **DGUV-Statistiken für die Praxis 2009**. Berlin: DGUV; 2010.
