# Supplementary material for: Bronchial asthma and COPD due to irritants in the workplace - an evidence-based approach
Source: J Occup Med Toxicol. 2012 Sep 26;7:19. doi: 10.1186/1745-6673-7-19 (PMC3508803; doi:10.1186/1745-6673-7-19)
Supplement: Additional file 2 — “Methodology” Selection criteria, information sources, strength of evidence. Table A: Data extraction and synthesis. Table B. Quality assessment of individual study. Table C - The revised Scottish Intercollegiate Guidelines Network (SIGN) grading system (modifications are given in italics) [87]. Table D The Royal College of General Practitioners (RCGP) three-star system [88] used by the British Occupational Health Research Foundation [3,574] (modifications are given in italics). [file 1745-6673-7-19-S2.docx]

Online Supplement „Methodology“

Table A Data extraction and synthesis

Relevant data from the chosen publications was added to an Excel spreadsheet as explained below. The following information was included in evidence tables (see Online supplement, Table E):

#### **Agent (latin)**

- CAS no.

#### **Publication [Reference]**

- first two authors and year of publication

**Strength of evidence**

- modified RCGP three-star system applied to the whole study group per agent, also considering methodological quality of the studies; i.e. down-grading if only questionnaire information was available

#### **Evidence grading, applied to individual study and considering study type**

- modified SIGN system

#### **Occupational exposed subjects studied**

- n: number of exposed workers examined

#### **Total no. of irritant-induced OA or COPD cases per agent**

- n: number of cases

#### **Irritant-induced OA or occupational COPD cases**

- n: number of cases and prevalence in %

**Diagnostic tests for irritant-induced OA or occupational COPD**

- Work-related symptoms, i.e. i) asthmatic symptoms or physician diagnosed occupational asthma or COPD (symptoms beginning after employment/exposure); ii) symptoms compatible with RADS (“+”)

Dependent on the study design, diagnosis of irritant-induced asthma or COPD was based on different diagnostic procedures according to the author. The definitions of confirmed cases were different. At least one of the following diagnostic tests had been used to confirm the diagnosis:

- LFT (lung function test, spirometry). Positive results(s) (obstructive ventilation pattern, i.e. number of cases with reduced FEV1/FVC according to Brändli et al. [[1](#_ENREF_1)] / all tested subjects; n/n; %
- SFT (serial lung function testing by spirometry or expiratory flow rate (PEF) measuring cross-shift (pre-, during, and post-shift) PEF). Positive result(s) (i.e. number of cases with fall in FEV1 or PEF during exercise of 15 or 20% according to the author) / all tested subjects; n/n; %

NSBHR: Positive result(s) (pathologically reduced PC20 (or PD20), i.e. number of cases with 8 or 16 mg/L according to the author) / all tested subjects; n/n; %

- SIC (specific inhalation challenge test). Positive result(s) (i.e. number of cases with fall in FEV1 (PEF) of 20% according to the author) / all tested subjects; n/n; %

reaction type:

i : immediate asthmatic reaction (0-60 min)

l: late asthmatic reaction (>60 min-24h)

d: dual asthmatic reaction (immediate and late)

#### **Remarks**

- occupation
- clinical tests:
  - immunological testing, such as skin-prick test, specific IgE
  - SFT: detailed description of serial PEF if > 1 shift (1 day), for example: serial PEF at work and off-work for x weeks
  - SIC: if tested agent not identical with first column of the Table 9 (adnex)

For example:

SIC with “component of agent” (chlorine)

4/10 SIC+ with “a second agent Y” (1 immediate, 3 late)

dose-response relationship, co-exposure to other irritant agents

1. Brändli O, Schindler C, Leuenberger PH, Baur X, Degens P, Kunzli N, Keller R, Perruchoud AP: **Re-estimated equations for 5th percentiles of lung function variables.** *Thorax* 2000, **55:**173-174.

Table B Quality assessment of individual study

The study quality was assessed with the help of the following check list.

**Check list of individual study**

**Design:**

- randomized/controlled clinical trial
- systematic review of cohort, case control or cross-sectional studies.
- cohort study (prospective/retrospective), longitudinal follow-up
- case control study
- cross-sectional study, survey
- review
- Case series, follow-up of cases, case reports

**Data collection**:

- prospective (may be selected retrospectively, but data is collected prospectively)
- unclear

**Description of population:**

- adequate (i.e. patients described in terms of age, gender and presenting signs and symptoms)
- inadequate

**Patient selection:**

- consecutive or random selection
- other - specify_______________
- not reported

**Selection bias:**

- yes (i.e. high risk of patient selection related to results)
- no

**Description of reference standard:**

- adequate (e.g. referral to standard SIC methodology, timing of lung function tests, referral to standard challenge dosages and methodology, involvement of an OA specialist for establishing the diagnosis; sufficient information to reproduce the test)
- inadequate

**Description of test** (referral to standard methodology; adequate: sufficient information to reproduce the test/ inadequate):

- case history (questionnaire) (adequate/inadequate)
- lung function test (adequate/inadequate)
- pulmonary function test (adequate/inadequate)
- non-specific bronchial challenge (adequate/inadequate)
- specific bronchial challenge (adequate/inadequate)
- other test (adequate/inadequate)

**Partial verification bias** (i.e. decision to perform the reference test is based upon the results of the test under examination; result of the test predicts patient moving on to the reference standard or vice versa):

- yes
- no

**Reporting results:**

- allows re-creation of contingency tables
- does not allow re-creation of contingency tables

**Confounding** (co-exposure; exposure not measured):

- yes
- no

**Precision of the study:**

- n (study size)
- OR
- P > 0.05

Online Resource „Methodology“

Table C - The revised Scottish Intercollegiate Guidelines Network (SIGN) grading system [[1](#_ENREF_1)] (modifications are given in italics)

| **SIGN grading** | **Description** |
| --- | --- |
| **1++** | High quality meta-analyses, systematic reviews of randomized controlled trials or randomized controlled trials with a very low risk of bias |
| **1+** | Well conducted meta-analyses, systematic reviews of randomized controlled trials or randomized controlled trials with a low risk of bias |
| **1-** | Meta-analyses, systematic reviews of randomized controlled trials or randomized controlled trials with a high risk of bias |
| **2++** | High quality systematic reviews of case-control or cohort studies. High quality case control or cohort studies with a very low risk of confounding, bias or chance and a high probability that the relationship is causal |
| **2+** | Well-conducted case control or cohort studies with a low risk of confounding, bias or chance and a moderate probability that the relationship is causal. Well-conducted systemic reviews of analytical cross-sectional studies |
| **2-** | Case control or cohort studies with a high risk of confounding, bias or chance and a significant risk that the relationship is not causal. Well-conducted analytical cross-sectional or longitudinal studies with low risk of confounding, bias or chance |
| ***3+*** | *Cross-sectional, longitudinal studies or surveys with high risk of confounding, bias or chance and a significant risk that the relationship is not causal, non-analytical studies with n ≥ 5 cases* |
| **3** | Non-analytic studies, e.g. case reports, case series |
| **4** | Expert opinion |

1. Harbour R, Miller J: **A new system for grading recommendations in evidence based guidelines**. *BMJ (Clinical research ed* 2001, **323**(7308):334-336.

Online Resource „Methodology“

Table D - The Royal College of General Practitioners (RCGP) three-star system [[1](#_ENREF_1)] used by the British Occupational Health Research Foundation [[2](#_ENREF_2), [3](#_ENREF_3)] (modifications are given in italics)

| **three stars grading^1^** | **Description** |
| --- | --- |
| *** | Strong evidence – provided by generally consistent findings in numerous, high quality scientific studies |
| ** | Moderate evidence – provided by generally consistent findings in fewer, smaller or lower quality scientific studies |
| **[*]^2^* | *Moderate evidence – provided by generally consistent findings in fewer, smaller or lower quality scientific studies, based on questionnaires or other weak evidence (clinical weakness (absence of LFT, PFT, SIC))* |
| * | Limited or contradictory evidence – provided by one scientific study or inconsistent findings in multiple scientific studies |
| *[*]^2^* | *Limited or contradictory evidence – provided by one scientific study based only on questionnaires or other weak evidence (clinical weakness (absence of LFT, PFT, SIC))* |
| *(*)^2^* | *Very limited or contradictory evidence – provided by at least three case reports, case series or one occupational disease statistic study with at least five asthma cases* |
| - | No scientific evidence – based on clinical studies, theoretical considerations and/or clinical consensus |

^1^ Further modified as detailed in Methods (Evidence-based approach), see main document

^2^ For modifications, see main document

1. The Royal College of General Practitioners RCGP: **The development and implementation of clinical guidelines. Report of the Clinical Guidelines Working Group**. In*.* London: RCGP; 1995.

2. Nicholson PJ, Cullinan P, Burge PS, Boyle C (eds.): **Occupational asthma: Prevention, identification & management: Systematic review & recommendations.** [**http://www.bohrf.org.uk/downloads/OccupationalAsthmaEvidenceReview-Mar2010.pdf**](http://www.bohrf.org.uk/downloads/OccupationalAsthmaEvidenceReview-Mar2010.pdf). London: British Occupational Health Research Foundation; 2010.

3. Newman Taylor AJ, Cullinan P, Burge PS, Nicholson P, Boyle C: **BOHRF guidelines for occupational asthma**. *Thorax* 2005, **60**(5):364-366.
